# The Association of G Protein-Coupled Estrogen Receptor (GPER) Polymorphisms with Ionizing Radiation Exposure in Healthcare Workers

**DOI:** 10.3390/jcm15124821

**Published:** 2026-06-21

**Authors:** Ünal Öztürk, Ergül Belge Kurutaş, Nuray Üremiş, Muhammed Mehdi Üremiş, Fatma Nur Özkömeç

**Affiliations:** 1 Department of Cardiology, Medical Faculty, Kahramanmaraş Sütçü İmam University, Kahramanmaraş 46050, Turkey; 2 Department of Medical Biochemistry, Medical Faculty, Kahramanmaraş Sütçü İmam University, Kahramanmaraş 46050, Turkey; 3 Department of Chemistry, Science Faculty, Kahramanmaraş Sütçü İmam University, Kahramanmaraş 46050, Turkey

**Keywords:** G protein-coupled receptor, single-nucleotide polymorphism, ionizing radiation, healthcare workers, in silico annotation

## Abstract

**Background/Objectives:** The G protein-coupled estrogen receptor (GPER) is known to interact with cellular stress responses and DNA damage pathways. Therefore, exposure to ionizing radiation may modulate the biological consequences of single-nucleotide polymorphisms in the *GPR30* gene. This study aims to evaluate the association between GPER polymorphisms and radiation sensitivity. **Methods:** The study included 50 healthcare workers exposed to ionizing radiation and 36 healthy individuals with no known occupational exposure to radiation. Genomic DNA was isolated and PCR products were purified using GeneAll kits. Genomic regions encompassing three GPER single-nucleotide polymorphisms (rs3808350, rs3808351, and rs11544331) were amplified by polymerase chain reaction (PCR), followed by DNA sequencing analysis using the BigDye Cycle Sequencing Kit. In addition, an in silico functional and clinical annotation of rs11544331 was performed using Ensembl VEP, SIFT, PolyPhen-2, AlphaMissense, CADD, UniProt, and ClinVar. **Results:** Genotypic, dominant, and allelic analyses revealed no significant association between radiation exposure and the rs3808350 or rs3808351 polymorphisms. In contrast, a statistically significant association was observed for rs11544331. The frequency of individuals carrying the CT and TT genotypes (CT + TT) was significantly higher in the ionizing radiation-exposed group compared with the control group (OR = 2.981; 95% CI: 1.106–7.904; *p* = 0.0241). In allelic analysis, the T allele was more prevalent in the exposed group and was significantly associated with radiation exposure (OR = 2.959; 95% CI: 1.282–6.606; *p* = 0.0110). In silico analysis confirmed that rs11544331 corresponds to the p.Pro16Leu substitution in GPER1; however, SIFT, PolyPhen-2, AlphaMissense, CADD, and ClinVar consistently indicated a tolerated, benign, likely benign, or low-deleteriousness profile. **Conclusions:** GPER-mediated stress responses and genetic polymorphisms may play a potential role in determining genetic susceptibility following exposure to ionizing radiation.

## 1. Introduction

Estrogens exert their cellular effects primarily through the classical nuclear estrogen receptors (ERα and ERβ) and the G protein-coupled estrogen receptor (GPER; formerly known as GPR30) [1,2]. These two signaling pathways differ markedly in terms of signal transduction kinetics, molecular mechanisms, and cellular outcomes. In the classical genomic pathway, the lipophilic estrogen molecule passively diffuses across the cell membrane and binds to ERα and ERβ receptors localized in the cytoplasm and/or nucleus [3]. Upon ligand binding, the activated estrogen–receptor complex translocates to the nucleus and regulates the transcription of target genes by binding to estrogen response elements on DNA [4]. This process is characterized by a genomic response that typically develops over hours to days and results in long-term cellular effects [5].

In contrast, estrogen signaling mediated through GPER is non-genomic, rapid, and dynamic in nature [6,7,8]. GPER is defined as a seven-transmembrane G protein-coupled estrogen receptor predominantly localized to the plasma membrane and the endoplasmic reticulum membrane [9]. Binding of estrogen to GPER leads to the activation of heterotrimeric G proteins (Gs, Gi, or Gq) [10]. Consequently, secondary messenger systems are activated, including increased cAMP production via adenylyl cyclase, intracellular Ca^2+^ mobilization, phospholipase C (PLC)-mediated IP_3_/DAG generation, protein kinase C (PKC) activation, and epidermal growth factor receptor (EGFR) transactivation [11]. As a result of these signaling events, cellular survival and proliferation-related pathways, such as MAPK/ERK and PI3K/AKT, are activated [10]. Therefore, the majority of studies in the current literature on GPER activation have focused primarily on cellular survival and proliferation pathways, particularly those associated with tumorigenesis [12,13]. These studies have reported that GPER activation promotes cell proliferation, suppresses apoptosis, and modulates cellular stress responses [13,14]. For instance, the selective GPER antagonists G15 and G36 have been shown in in vitro cancer models to suppress GPER-mediated proliferative and survival signaling, resulting in effects associated with apoptosis induction and cell cycle arrest [15,16]. Moreover, ionizing radiation is known to induce the generation of reactive oxygen species (ROS), leading to oxidative stress and various forms of DNA damage, including single- and double-strand breaks [17]. These DNA lesions activate key DNA damage response pathways, particularly the ataxia telangiectasia mutated (ATM) and ATM- and Rad3-related (ATR) signaling cascades, which regulate cell cycle arrest, DNA repair, and apoptosis [18]. Previous studies have focused on the role of GPER in the regulation of cellular stress responses, survival signaling pathways, and apoptosis; however, the relationship between ionizing radiation exposure and the biological consequences of GPER single-nucleotide polymorphisms has not yet been investigated. Considering the central role of DNA damage in the biological effects of ionizing radiation, genetic variations in the GPER gene may have functional relevance in determining individual responses to radiation exposure. Therefore, this study aimed to evaluate the relationship between GPER polymorphisms and radiation sensitivity.

The selection of single-nucleotide polymorphisms (SNPs) in the GPER gene (rs3808350, rs3808351, and rs11544331) was based on a combined evaluation of the existing literature, potential functional relevance, and population genetic considerations. These polymorphisms have been investigated in previous studies across various disease contexts and have been reported to be associated with disease susceptibility and diverse clinicopathological and phenotypic characteristics [19,20,21]. In particular, rs3808350 and rs3808351 are located in the promoter region of the GPER gene and may influence gene expression, whereas the rs11544331 variant has been suggested to affect receptor function and intracellular signaling processes [22,23]. In addition, these SNPs were selected because they exhibit sufficient allele frequency distribution in human populations, allowing for reliable comparisons between groups to be made and providing adequate statistical power for association analyses [19,24]. Taken together, these variants were considered biologically plausible candidates for evaluating genetic susceptibility to ionizing radiation exposure.

## 2. Patients and Methods

### 2.1. Study Population

The study included healthcare workers employed at state and university hospitals in the provinces of Kahramanmaraş, Mersin, Adana, and Hatay, who worked in coronary angiography laboratories and were exposed to ionizing radiation due to the fluoroscopy/scopy systems used during coronary angiography procedures. The study was approved by the Kahramanmaraş Sütçü İmam University Medical Research Ethics Committee under protocol number 2021/38-309. The patient group consisted of 50 individuals aged 30–55 years who had been working in the field of invasive cardiology (coronary angiography) for at least 10 years. Approximately 80% of the participants routinely used personal dosimeters as part of occupational radiation monitoring. However, individual cumulative or annual radiation dose records were not accessible for all participants; therefore, quantitative dose-based exposure assessment could not be performed. Individuals with chronic systemic diseases, with a history of malignancy, who were active smokers, or those who were pregnant, breastfeeding, or postmenopausal were excluded from the study. The control group comprised 36 healthy individuals with no occupational exposure to ionizing radiation. Written informed consent was obtained from all participants prior to their inclusion in the study, in accordance with the principles of the Declaration of Helsinki.

In Türkiye, occupational exposure to ionizing radiation is regulated by the Nuclear Regulatory Authority (NDK), and healthcare workers are routinely monitored using personal dosimeters. The annual effective dose limit is defined as 20 mSv/year averaged over 5 years, with a maximum of 50 mSv in any single year. In the present study, although most participants reported regular dosimeter use, individual annual or cumulative dose records (mSv/year) were not consistently accessible. Therefore, quantitative dose-based exposure assessment could not be performed, and exposure was defined based on occupational history (≥10 years in interventional cardiology laboratories), representing chronic low-dose radiation exposure.

### 2.2. DNA Isolation from Whole-Blood Samples

Genomic DNA was isolated from whole-blood samples collected in anticoagulant (EDTA) tubes, following the manufacturer’s instructions (GeneAll Exgene Blood SV Mini, GeneAll Biotechnology Co., Ltd., Seoul, 05729, Republic of Korea). Briefly, cells were lysed using a lysis buffer in the presence of Proteinase K. DNA was then bound to a silica membrane column by the addition of ethanol. The column was washed sequentially with wash buffers, and genomic DNA was eluted under appropriate conditions. The isolated DNA samples were subsequently used for PCR analyses.

### 2.3. PCR Amplification and Purification

Polymerase chain reaction (PCR) was performed to identify the single-nucleotide polymorphisms (SNPs). PCR amplification was carried out using the Wizbio WizPure™ PCR 2X Master Mix kit (WIZBIO Solutions Inc., Seongnam-si, Republic of Korea). The sequences and characteristics of the primers used in PCR are presented in Table 1. Genomic regions encompassing the selected polymorphisms in the GPER gene (rs3808350, rs3808351, and rs11544331) were amplified by polymerase chain reaction (PCR) using specific primer pairs. Amplicon sizes and genomic binding positions of each primer pair were determined using the NCBI Primer-BLAST tool (https://www.ncbi.nlm.nih.gov/tools/primer-blast/, accessed on 4 February 2026) based on the Homo sapiens reference genome (GRCh38). Specificity analysis was performed against the RefSeq genomic database, and only uniquely matching primer pairs were considered. The coordinates of primer binding sites were identified, and expected PCR product sizes were calculated based on the distance between forward and reverse primer binding regions.

The reaction mixture was prepared using PCR 2X Master Mix, forward primer (10 μM), reverse primer (10 μM), nuclease-free water, and DNA template. The PCR cycling conditions are listed in Table 2. All PCRs were performed under a unified set of cycling conditions following an initial optimization step. Primer pairs for each SNP were designed to have comparable melting temperatures (Tm) and GC content, allowing for the use of a single annealing temperature (67 °C) across all reactions. The use of uniform PCR conditions minimized inter-assay variability and improved reproducibility across samples.

Amplification was performed on an Applied Biosystems™ ProFlex (Thermo Fisher Scientific, Waltham, MA, USA) thermal cycler and verified by 2% agarose gel electrophoresis. PCR products were purified using the GeneAll^®^ Expin™ Gel SV gel extraction kit (GeneAll Biotechnology Co., Ltd., Seoul, 05729, Republic of Korea), following the manufacturer’s protocol. Briefly, gel slices were completely dissolved in the appropriate buffer and transferred to a silica membrane column. DNA bound to the column was washed sequentially to remove impurities and finally eluted using the elution buffer. The purified PCR products were subsequently used for DNA sequencing reactions.

### 2.4. DNA Sequencing Reaction and Purification

Sequencing reactions were performed using the BigDye™ Terminator v3.1 Cycle Sequencing Kit (Thermo Fisher Scientific, Waltham, MA, USA). The sequencing reaction mixture was prepared using BigDye™ Terminator v3.1 Ready Reaction Mix, Sequencing Buffer, forward or reverse primer (3.2 μM), RNase/DNase-free water, and the purified DNA template. The thermal cycling conditions used for sequencing are presented in Table 3.

All sequencing reactions were carried out on an Applied Biosystems™ ProFlex thermal cycler. Following the sequencing reaction, the products were purified using Sephadex gel filtration. The eluate obtained after purification was used as the purified sequencing product for subsequent analyses.

### 2.5. Capillary Electrophoresis

Capillary electrophoresis analysis was performed using the ABI 3500 Genetic Analyzer (Thermo Fisher Scientific, Waltham, MA, USA). Purified sequencing products were mixed with formamide, loaded into 96-well plates, and subjected to electrophoresis. The resulting data were analyzed based on the electropherograms generated by the instrument.

### 2.6. In Silico Functional and Clinical Annotation of Rs11544331

To address the potential functional relevance of the rs11544331 polymorphism, an in silico functional and clinical annotation analysis was performed. The variant was mapped to the human GPER1 protein sequence using Ensembl Variant Effect Predictor (VEP; GRCh38/hg38; Ensembl, Cambridge, UK) [25], UniProt variant annotation (Q99527; UniProt, Geneva, Switzerland) [26], AlphaMissense (available at https://alphamissense.hegelab.org, accessed on 1 February 2026) [27], CADD (Combined Annotation-Dependent Depletion; GRCh38-v1.7) [28], SpliceAI annotations obtained from the CADD output (available at https://spliceailookup.broadinstitute.org/, accessed on 1 February 2026), and ClinVar (available at https://www.ncbi.nlm.nih.gov/clinvar/, accessed on 1 February 2026) [29,30]. The corresponding gene, transcript, coding consequence, codon change, amino acid substitution, protein position, allele frequency, and clinical annotation were recorded. Ensembl VEP was used to determine the molecular consequence of rs11544331 and to obtain SIFT and PolyPhen-2 predictions [31,32]. UniProt was used to confirm the protein-level annotation of the variant in GPER1_HUMAN [26]. AlphaMissense was used to assess the predicted pathogenicity of the corresponding missense substitution. CADD was used to evaluate the integrated deleteriousness score of the variant, including raw and PHRED-scaled scores. SpliceAI annotations obtained from the CADD output were recorded to assess whether rs11544331 was predicted to have any splice-altering effect. ClinVar was used to evaluate available clinical classification, review status, variation accession, and reported allele frequency. The rs11544331 variant was specifically evaluated as NM_001098201.3(GPER1):c.47C>T, corresponding to the p.Pro16Leu substitution in GPER1. Prediction outputs were interpreted according to the classification criteria provided by each database or algorithm. The aim of this analysis was not to establish pathogenicity definitively but to determine whether the observed amino acid substitution is predicted to be deleterious or benign and to provide biological context for the genetic association findings.

### 2.7. Statistical Analysis

Baseline demographic, lifestyle, and laboratory variables (sex, age, mobile phone use, computer use, BMI, urea, creatinine, triglycerides, total cholesterol, HDL cholesterol, and LDL cholesterol) were summarized as mean ± standard deviation (SD) for continuous variables and as number (percentage) for categorical variables. Comparisons between the exposed group and controls were performed using Student’s unpaired *t*-test for continuous variables and the chi-square test (or Fisher’s exact test when appropriate) for categorical variables.

Genotype and allele frequencies were calculated by direct counting. Allele counts were derived from genotype counts, and allele frequencies were calculated based on the total number of alleles (2N) in each group. Differences in genotype distributions between ionizing radiation-exposed healthcare workers and non-exposed controls were evaluated using the chi-square (χ^2^) test or Fisher’s exact test, as appropriate (Fisher’s exact test was applied when expected cell counts were <5 or when a genotype category contained zero counts). Hardy–Weinberg equilibrium (HWE) was assessed in the control group using a chi-square goodness-of-fit test by comparing observed genotype counts with expected counts calculated from allele frequencies. In addition, an exact test [33] was performed to account for the relatively small sample size and low expected genotype counts, and both *p* values are reported. Associations with occupational ionizing radiation exposure were estimated by odds ratios (ORs) with 95% confidence intervals (CIs) using 2 × 2 contingency tables under dominant (variant carriers vs. wild-type homozygotes) and allelic (minor vs. major allele) models. In this design, the OR quantified the strength of the association between a given genotype or allele and group membership (exposed vs. control): an OR > 1 indicated over-representation of the variant carrier state or minor allele in the exposed group relative to the controls, whereas an OR < 1 indicated under-representation. Because exposure status was defined occupationally rather than as a biological outcome, ORs were interpreted as measures of genotype–exposure association (variant enrichment) and not as causal estimates of the risk of radiation exposure. The calculated odds ratios and corresponding 95% confidence intervals were additionally visualized using a forest plot to facilitate interpretation and comparison across genetic models. Because of the relatively small and unequal group sizes, statistical significance for the 2 × 2 dominant and allelic models was assessed using Barnard’s unconditional exact test (two-sided) [34]. All tests were two-tailed, and *p* values < 0.05 were considered statistically significant. All analyses were performed using GraphPad Prism 10 (GraphPad Software, San Diego, CA, USA), except for Barnard’s unconditional exact test, which was computed using a dedicated online calculator [35].

## 3. Results

### 3.1. Clinical Characteristics

The clinical and demographic characteristics of healthcare workers exposed to ionizing radiation and the control group are presented in Table 4. The exposed group included 18 females and 32 males, whereas the control group comprised 12 females and 24 males. The mean age was 39.83 ± 8.05 years in the exposed group and 36.21 ± 7.31 years in the control group. Daily mobile phone usage was 13.3 ± 3.1 h in the exposed group and 12.45 ± 1.6 h in the control group, while daily computer usage was 7.5 ± 4.3 h and 6.4 ± 3.6 h, respectively. Body mass index (BMI) values were 25.31 ± 4.16 kg/m^2^ in the exposed group and 24.67 ± 4.56 kg/m^2^ in the control group. Biochemical parameters showed that urea levels were 28.44 ± 5.4 mg/dL in the exposed group and 30.41 ± 5.12 mg/dL in the control group, while creatinine levels were 0.84 ± 0.19 mg/dL and 0.83 ± 0.20 mg/dL, respectively. Analysis of the lipid profile revealed similar distributions of triglycerides, total cholesterol, HDL cholesterol, and LDL cholesterol between the groups. Triglyceride levels were 208.4 ± 103.1 mg/dL in the exposed group and 210.7 ± 106.4 mg/dL in the control group. Total cholesterol, HDL cholesterol, and LDL cholesterol levels in the exposed group were 155.1 ± 39.4 mg/dL, 36.1 ± 10.1 mg/dL, and 79.9 ± 28.5 mg/dL, respectively, compared with 152.7 ± 42.9 mg/dL, 38.5 ± 9.6 mg/dL, and 75.8 ± 30.2 mg/dL in the control group. Statistical analysis revealed no statistically significant differences between the exposed and control groups for any of the evaluated clinical, demographic, or biochemical parameters (*p* > 0.05).

Values are presented as mean ± standard deviation (SD) unless otherwise indicated; sex is presented as number. Group comparisons were performed using Student’s unpaired *t*-test for continuous variables and the chi-square test (or Fisher’s exact test when appropriate) for categorical variables. No statistically significant differences were observed between the exposed group and controls for any variable (all *p* > 0.05).

### 3.2. Rs3808350 Genotype and Allele Distributions

The genotype and allele distributions of the rs3808350 polymorphism in healthcare workers exposed to ionizing radiation and the control group are presented in Table 5. In the exposed group, the frequencies of the AA, AG, and GG genotypes were 38%, 52%, and 10%, respectively, whereas in the control group, they were 22.22%, 66.66%, and 11.11%, respectively. No statistically significant difference was observed between the groups in terms of genotype distribution (χ^2^ test, *p* > 0.05). Under the dominant model (AG + GG vs. AA), the frequency of AG + GG genotypes was 62% in the exposed group and 77.78% in the control group. Comparison under this model revealed no significant association between the rs3808350 polymorphism and exposure to ionizing radiation (OR = 0.4662; 95% CI: 0.1736–1.167; *p* = 0.1357). Allele frequencies showed that G and A alleles were distributed at 36% and 64% in the exposed group and 44.44% and 55.56% in the control group, respectively. Analysis under the allelic model also revealed no statistically significant difference between the groups (OR = 0.7031; 95% CI: 0.3852–1.280; *p* = 0.3330).

### 3.3. Rs3808351 Genotype and Allele Distributions

The genotype and allele distributions of the rs3808351 polymorphism in the ionizing radiation-exposed group and the control group are presented in Table 6. In the exposed group, the frequencies of the GG, GA, and AA genotypes were 50%, 42%, and 8%, respectively, whereas in the control group, they were 44.44%, 44.44%, and 11.11%, respectively. No statistically significant differences in genotype distributions were observed between the groups (χ^2^ test, *p* > 0.05). Under the dominant model (GA + AA vs. GG), no significant association was found between rs3808351 and exposure to ionizing radiation (OR = 0.8000; 95% CI: 0.3285–1.893; *p* = 0.6319). Similarly, allelic analysis (A vs. G) revealed no statistically significant difference between the groups (OR = 0.8169; 95% CI: 0.4196–1.550; *p* = 0.6026).

### 3.4. Rs11544331 Genotype and Allele Distributions

The genotype and allele distributions of the rs11544331 polymorphism are presented in Table 7. In the ionizing radiation-exposed group, the frequencies of the CC, CT, and TT genotypes were 54%, 38%, and 8%, respectively, whereas in the control group, the CC and CT genotypes were observed at 77.78% and 22.22%, respectively. A significant difference in genotype distribution was observed between the groups (*p* = 0.0367). Under the dominant model (CT + TT vs. CC), individuals carrying the T allele were significantly more frequent in the exposed group compared with the control group (OR = 2.981; 95% CI: 1.106–7.904; *p* = 0.0241). In the allelic analysis, the T allele frequency was 27% in the exposed group and 11.11% in the control group, showing a significant association between the T allele and exposure to ionizing radiation (OR = 2.959; 95% CI: 1.282–6.606; *p* = 0.0110). Taken together, these results indicate that the odds of carrying the CT + TT genotype, and of carrying the T allele, were each approximately three-fold higher among radiation-exposed workers than among controls.

### 3.5. Genotype and Allele Distributions

**Figure 1:**
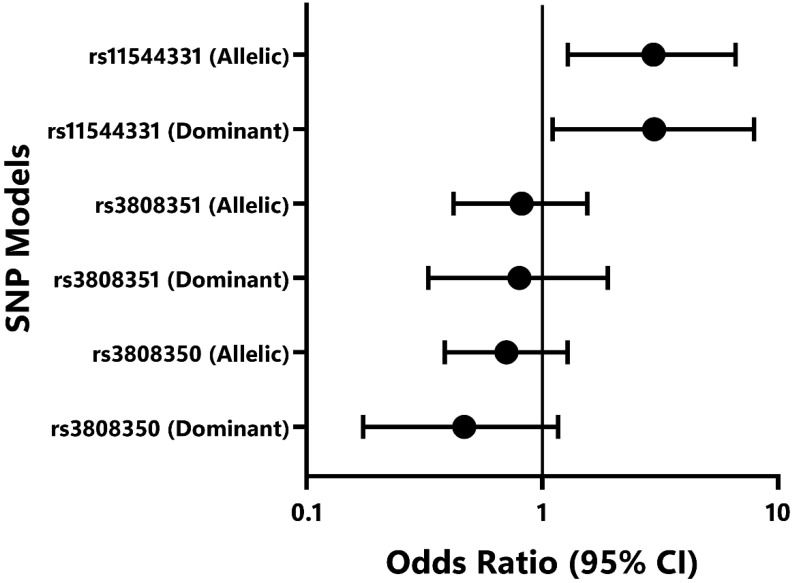
Forest plot of odds ratios for GPER1 polymorphisms and occupational ionizing radiation exposure. Odds ratios (ORs) and 95% confidence intervals (CIs) for the association between GPER1 polymorphisms (rs3808350, rs3808351, and rs11544331) and occupational ionizing radiation exposure are presented under dominant and allelic genetic models. ORs were calculated using 2 × 2 contingency tables comparing exposed healthcare workers (cases) and non-exposed individuals (controls). The vertical reference line at OR = 1 indicates no association. Values greater than 1 indicate higher odds of the variant carrier state (dominant model) or minor allele (allelic model) in the exposed group relative to controls (enrichment), whereas values less than 1 indicate the opposite. As exposure was defined occupationally, these odds ratios reflect the strength of the genotype–exposure association rather than a causal risk of radiation exposure. Among the evaluated polymorphisms, rs11544331 demonstrated a significant association with occupational radiation exposure under both dominant and allelic models, while rs3808350 and rs3808351 showed no statistically significant associations.

The three GPER gene polymorphisms (rs3808350, rs3808351, and rs11544331) were evaluated in healthcare workers exposed to ionizing radiation and the control group. No statistically significant associations were observed between the groups for rs3808350 and rs3808351 in genotypic, dominant, or allelic comparisons (Table 5 and Table 6). In contrast, a statistically significant association was found between the rs11544331 polymorphism and occupational exposure to ionizing radiation (Table 7). These associations are further illustrated in a forest plot (Figure 1), showing the odds ratios (ORs) and 95% confidence intervals (CIs) for all polymorphisms under dominant and allelic models. Genotype distributions of all three loci in the control group did not significantly deviate from Hardy–Weinberg equilibrium (rs3808350: *p* = 0.0685; rs3808351: *p* > 0.9999; rs11544331: *p* = 0.7549). Exact test analysis yielded results consistent with the chi-square test and confirmed no significant deviation from Hardy–Weinberg equilibrium for any of the studied polymorphisms (rs3808350: *p* = 0.0942; rs3808351: *p* = 1.0000; rs11544331: *p* = 0.7526) (Table 8).

Hardy–Weinberg equilibrium (HWE) was assessed in the control group using both a chi-square goodness-of-fit test and an exact test [33] by comparing observed and expected genotype frequencies.

### 3.6. In Silico Functional and Clinical Annotation of Rs11544331

Ensembl VEP, UniProt, AlphaMissense, CADD, and ClinVar annotations confirmed that rs11544331 corresponds to a missense variant in the GPER1 gene. The variant was annotated as NM_001098201.3(GPER1):c.47C>T and results in a p.Pro16Leu substitution at amino acid position 16 of the GPER1 protein. The codon change was identified as CCA>CTA, and the genomic location was reported as NC_000007.14:g.1091775C>T, corresponding to GRCh38 position 7:1091775. Ensembl VEP classified the variant as a moderate-impact missense variant, consistent with its non-synonymous amino acid substitution. SIFT predicted the p.Pro16Leu substitution as tolerated, with a score of 0.19, whereas PolyPhen-2 predicted it as benign, with a score of 0.000. AlphaMissense also classified the variant as likely benign, with a pathogenicity score of 0.0743. CADD analysis yielded a raw score of 0.113797 and a PHRED score of 1.654, indicating a low predicted deleteriousness. In addition, SpliceAI scores were 0.000 for acceptor gain, acceptor loss, donor gain, and donor loss, suggesting no predicted splice-altering effect. Consistently, ClinVar classified the germline variant as benign. However, this ClinVar classification was based on a single submitter with criteria provided, and no data were available regarding somatic clinical impact or oncogenicity. Taken together, these findings indicate that rs11544331 is not predicted to be a highly deleterious pathogenic variant by current in silico and clinical annotation resources. Nevertheless, because rs11544331 produces a true non-synonymous amino acid substitution in GPER1, its potential biological relevance may be related to subtle modulatory effects on receptor processing, localization, ligand responsiveness, or downstream signaling rather than a classical pathogenic mechanism. The complete annotation summary is presented in Table 9.

## 4. Discussion

GPER has been increasingly recognized as an important regulator of immune cell function and inflammatory signaling [36]. Experimental studies have demonstrated that GPER is expressed in various immune cells, including monocytes, macrophages, and lymphocytes, where it modulates cytokine production and cellular activation [36]. Activation of GPER has been shown to influence key inflammatory pathways, including the regulation of IL-6, TNF-α, and IL-1β, as well as intracellular signaling cascades such as MAPK/ERK and PI3K/Akt pathways [11]. Moreover, GPER-mediated signaling has been linked to the modulation of oxidative stress by affecting the balance between ROS production and antioxidant defense mechanisms [37]. In the context of ionizing radiation exposure, which is known to induce persistent oxidative stress and immune activation even at low doses, alterations in GPER function due to genetic polymorphisms may have important biological consequences. Chronic radiation exposure has been associated with sustained low-grade inflammation, dysregulated cytokine profiles, and increased oxidative damage in circulating immune cells [38]. Therefore, genetic variations such as rs11544331 may contribute to interindividual differences in immune response and oxidative stress regulation in exposed healthcare workers.

In a previous study conducted by our research group, significantly increased oxidative and nitrosative stress was demonstrated in 100 radiodiagnostic laboratory personnel occupationally exposed to ionizing radiation compared to controls [39]. Specifically, decreased catalase (CAT) activity, along with increased levels of malondialdehyde (MDA), nitric oxide (NO), and 3-nitrotyrosine (3-NT), was observed in the exposed group. These findings indicate a marked disruption in oxidant–antioxidant balance and support the presence of persistent ROS-mediated cellular stress in radiation-exposed healthcare workers. Considering that oxidative stress is a key upstream mechanism linking ionizing radiation to DNA damage, inflammation, and immune dysregulation, these results provide indirect functional support for the biological relevance of the GPER polymorphisms identified in the present study.

In this study, the distributions of the GPER gene polymorphisms rs3808350, rs3808351, and rs11544331 were evaluated in healthcare workers occupationally exposed to ionizing radiation, and the rs11544331 variant was found to be significantly associated with radiation exposure. These findings suggest that the GPER signaling pathway may play a potential role in cellular stress responses induced by ionizing radiation. While GPER polymorphisms have been previously linked to various hormone-related cancers and endocrine disorders, studies examining these genetic variants in the context of ionizing radiation exposure are extremely limited. In this regard, our study represents the first investigation of a possible association between GPER polymorphisms and occupational radiation exposure.

Low-dose ionizing radiation exposure remains an important occupational health concern for healthcare workers. Chronic exposure, even within legal dose limits, contributes to oxidative stress and DNA damage [40]. A study conducted on hospital workers with occupational exposure to low-dose ionizing radiation reported increased activities of CuZn-SOD and CAT, elevated MDA levels, and a significant rise in micronucleus frequency. It was suggested that chronic low-dose radiation may enhance oxidative stress, thereby adversely affecting chromosomal integrity [41]. In a study of radiology personnel with long-term occupational exposure to low-dose X-rays, significant increases in micronucleus frequency and lipid peroxidation, along with decreased activities of antioxidant enzymes, were observed. These biological effects were reported to be associated with duration of employment, cumulative radiation dose, and daily patient workload, highlighting that chronic low-dose radiation may promote genomic instability through oxidative stress [42]. In another study of healthcare workers occupationally exposed to ionizing radiation, superoxide (O_2_•^−^) levels were found to be significantly elevated, with a selective increase observed in proinflammatory cytokines, particularly IL-6, IL-1α, and MIP-1α [43]. These studies indicate that low-dose ionizing radiation can leave a trace of DNA damage in peripheral blood lymphocytes and increase oxidant levels in plasma [41,42,43]. Therefore, previous studies have focused on the effects of ionizing radiation exposure on stress markers and systemic stress responses. Research on GPER signaling has demonstrated that GPER regulates both rapid (non-genomic) and genomic effects across major organs, indicating that its activation or inhibition is directly associated with the pathogenesis of various diseases [37]. Studies in cardiovascular, orthopedic, endocrinological, neurological, and oncological diseases, as well as in female-related conditions such as breast cancer and polycystic ovary syndrome, suggest that mutations in the GPER gene may be associated with disease pathogenesis and clinical phenotypes [19,21,23,44,45,46,47]. In a previous case–control study, the rs3808350, rs3808351, and rs11544331 polymorphisms of the GPER gene were not directly associated with the development of breast cancer. However, these variants were reported to show significant correlations with clinicopathological parameters such as tumor size, histological grade, lymph node status, and progesterone receptor (PR) expression. Notably, the rs3808351 and rs11544331 alleles were particularly associated with less aggressive tumor characteristics and PR positivity [19]. In a study on testicular carcinogenesis, the loss of the homozygous wild-type (GG) genotype at the rs3808350 and rs3808351 polymorphisms located in the promoter region of the GPER gene was reported to occur significantly more frequently in seminoma cases, whereas it was not observed in non-seminomatous testicular germ cell tumors. Furthermore, it was suggested that genetic predispositions associated with alterations in GPER expression may influence the development of testicular cancer [21]. In a study conducted on hypertensive patients, in addition to the commonly reported single-nucleotide polymorphisms of GPER, the functional significance of the GPER P16L variant was investigated. The P16L variant was reported to be a hypofunctional allele and was associated with increased blood pressure in women carrying this allele [23]. In this study, three GPER polymorphisms (rs3808350, rs3808351, and rs11544331) were evaluated in individuals occupationally exposed to low-dose ionizing radiation. Genotype distributions in the control group were generally consistent with Hardy–Weinberg equilibrium; however, given the relatively small sample size, these findings should be interpreted with caution. Importantly, the polymorphism showing a significant association (rs11544331) was fully consistent with HWE in both chi-square and exact test analyses, supporting the validity of the observed odds ratios, and rs3808350 likewise showed no significant deviation from HWE in either the chi-square or the exact test. Our findings indicate that the association with occupational radiation exposure was observed for the rs11544331 polymorphism, whereas the rs3808350 and rs3808351 variants showed no significant associations in genotypic, dominant, or allelic models. Quantitatively, the T allele and the CT + TT genotype were each enriched approximately three-fold in the exposed group; consistent with the study design, this odds ratio reflects the strength of the genotype–exposure association rather than a causal risk conferred by the variant. The observed association of the rs11544331 variant with radiation exposure may be biologically supported by previous studies suggesting that certain GPER polymorphisms can influence receptor localization and downstream signaling pathways. For instance, experimental evidence has indicated that specific variants may alter intracellular trafficking and functional behavior of the receptor [48]. However, as the study conducted by our team did not include localization analyses, the functional implications of the downstream pathways mediated by GPER polymorphisms could be elucidated through further research. Studies investigating the association of GPER gene polymorphisms with clinical cases have reported varying genotype and allele distributions. The heterogeneity observed across different phenotypes related to GPER polymorphisms likely arises from disease-specific pathophysiological mechanisms exerting distinct effects on the regulation of the GPER gene. Consequently, disease-dependent alterations in receptor activity may influence cellular responses to stimuli and contribute to increased phenotypic heterogeneity.

To directly address the functional relevance of the statistically significant rs11544331 finding, we performed an in silico functional and clinical annotation of this variant. The analysis confirmed that rs11544331 corresponds to the p.Pro16Leu substitution in GPER1. Although this substitution was classified as a moderate-impact missense variant by Ensembl VEP due to the amino acid change, pathogenicity prediction tools consistently suggested a benign, tolerated, or likely benign effect. Specifically, SIFT predicted the variant as tolerated, PolyPhen-2 predicted it as benign, AlphaMissense classified it as likely benign, and ClinVar annotated the germline variant as benign. CADD analysis further supported this interpretation, with a low PHRED score of 1.654, indicating that the variant is not predicted to have high deleteriousness. These findings are important for interpreting the biological meaning of the observed association. The significant association between rs11544331 and occupational ionizing radiation exposure should not be interpreted as evidence that p.Pro16Leu is a highly deleterious monogenic mutation. Rather, the association may reflect a modest modulatory effect of this non-synonymous substitution, a population-specific genetic association, or linkage disequilibrium with nearby regulatory variation affecting GPER1 expression or signaling. This interpretation is biologically plausible because chronic exposure to ionizing radiation is associated with oxidative stress, inflammatory activation, and DNA damage response pathways, all of which may interact with GPER1-mediated cellular stress signaling. Notably, Feldman et al. previously demonstrated that the P16L variant represents a hypofunctional allele associated with im-paired receptor glycosylation and altered subcellular localization, leading to reduced downstream signaling capacity [23]. Furthermore, Pupo et al. showed that this polymorphism repurposes GPER1 toward transcription factor-like paracrine signaling activity, independent of its membrane-associated canonical role [48]. These experimental findings indicate that the functional consequence of rs11544331 may operate through post-translational and intracellular trafficking mechanisms not fully captured by sequence conservation-based prediction tools. Therefore, the present findings suggest that rs11544331 may act as a potential susceptibility-associated marker rather than a directly pathogenic variant. Under chronic oxidative and genotoxic stress conditions, even subtle differences in receptor maturation, subcellular localization, ligand responsiveness, or downstream signaling efficiency may contribute to interindividual variability in radiation response. However, because the in silico predictions did not indicate a strongly damaging effect, further experimental studies are required to determine whether p.Pro16Leu directly affects GPER1 protein expression, glycosylation, receptor trafficking, or stress-response signaling in radiation-exposed cells.

**Figure 2:**
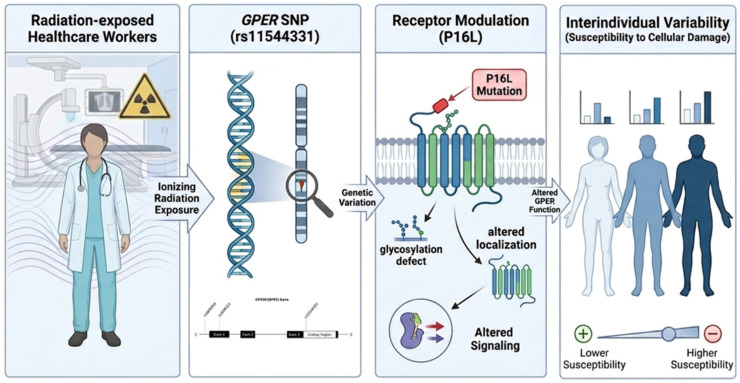
Conceptual model illustrating the potential relationship between occupational ionizing radiation exposure and GPER rs3808350, rs3808351, and rs11544331 polymorphisms in healthcare workers. Chronic low-dose occupational exposure to ionizing radiation in healthcare workers may interact with individual genetic background, including the GPER rs11544331 (p.Pro16Leu) variant. In the present study, rs11544331 was the only evaluated GPER polymorphism significantly associated with radiation exposure, whereas rs3808350 and rs3808351 showed no significant associations. Based on previous functional studies and in silico annotations, the p.Pro16Leu substitution may be associated with subtle changes in GPER processing, localization, or signaling efficiency rather than a strongly pathogenic effect. Such modulatory differences may contribute to interindividual variability in biological responses or susceptibility to radiation-related cellular damage. This figure represents a hypothesis-generating framework based on the observed genetic association and existing functional evidence, without implying direct mechanistic causality. This figure was created with FigureLabs (available at https://www.figurelabs.com/, accessed on 4 February 2026).

Taken together, genetic variation in GPER, particularly the rs11544331 polymorphism, may influence receptor function, intracellular signaling efficiency, or receptor localization, thereby altering the balance between DNA damage and repair processes under conditions of chronic radiation exposure (Figure 2). Accordingly, altered GPER signaling may affect the activation threshold or efficiency of downstream pathways involved in oxidative stress response and DNA repair, ultimately contributing to interindividual differences in susceptibility to radiation-induced cellular damage. Although the present study did not directly assess functional outcomes, the observed association between rs11544331 and radiation exposure may reflect underlying differences in cellular signaling dynamics and DNA damage response efficiency, supporting a potential mechanistic link between GPER polymorphisms and radiation sensitivity.

## 5. Conclusions

In this study, GPER gene polymorphisms were evaluated in healthcare workers with occupational exposure to ionizing radiation, and a significant association was identified between the rs11544331 variant and radiation exposure. The findings suggest that GPER-mediated signaling pathways may play a potential role in determining genetic susceptibility to cellular stress and DNA damage induced by ionizing radiation. This study can be considered exploratory due to the relatively modest sample size. Therefore, the findings should be interpreted with caution and require validation in larger, independent cohorts. Considering the limited sample size of the study, further research is needed to validate these results in larger populations. Large-scale, multicenter cohort studies including participants exposed to various sources of ionizing radiation (e.g., fluoroscopy, computed tomography, radiotherapy, or nuclear medicine procedures) may help to clarify the relationship between GPER polymorphisms and radiation sensitivity. In addition, the relatively small sample size and observational design may limit the generalizability of the findings to broader populations and preclude definitive causal inferences. Nevertheless, the consistency of the observed association provides a meaningful basis for future mechanistic and large-scale studies.

## 6. Limitations

The relatively small control sample size may have limited the statistical power and robustness of the Hardy–Weinberg equilibrium analysis. Therefore, these results should be interpreted with caution and validated in larger cohorts. In addition, although an in silico functional and clinical annotation of rs11544331 was incorporated into the revised manuscript, the present study did not experimentally validate the functional consequences of the p.Pro16Leu substitution. Therefore, future studies should investigate whether this variant affects GPER1 expression, receptor maturation, glycosylation, subcellular localization, ligand responsiveness, oxidative stress signaling, and DNA damage response pathways in radiation-exposed cells. Another limitation is the lack of stratification according to the intensity and duration of ionizing radiation exposure. Due to the absence of comprehensive dosimetric data and the limited sample size, exposure was assessed using a binary classification (exposed vs. non-exposed). Despite these limitations, the study provides novel evidence on the potential association between GPER polymorphisms and radiation sensitivity, offering a basis for future large-scale and mechanistic investigations.

## Figures and Tables

**Table 1:** Primers and sequences used in PCR.

Primer	Forward and Reverse Sequence	Synthesizing Firm	Amplicon Size (bp)
rs3808350	F: CCAGATGCCCCGATCCGCTGACTCT R: CCTCGGGACCTGCTGGGGCTCA	Oligomer	154
rs3808351	F: GGGGGAGCCCACGCAGGTCTCTG R: CCTCCCGCGCTCGCTGCAGAA	Oligomer	121
rs11544331	F: ACGCCCGCCGGACGAGCAC R: GGTCCGCCACCGCCAGGTTGA	Oligomer	182

**Table 2:** Conventional PCR conditions.

PCR Step	Temp (°C)	Time	Cycle
Initial Denaturation	95	5 min	1
Denature	95	30 s	30
Anneal	67	30 s	
Extension	72	30 s	
Final Extension	72	5 min	1
Cooling	4	-	1

**Table 3:** DNA sequencing reaction conditions.

PCR Step	Temp (°C)	Time	Cycle
Initial Denaturation	95	1 min	1
Denature	95	10 s	30
Anneal	50	5 s	
Extension	60	4 s	
Cooling	4	-	1

**Table 4:** Baseline demographic, lifestyle, and laboratory characteristics of ionizing radiation-exposed healthcare workers and non-exposed controls.

Variable	Case	Control	*p* Value
Sex (female/male), *n*	18/32	12/24	-
Age (years)	39.83 ± 8.05	36.21 ± 7.31	0.06
Mobile phone use (hours/day)	13.3 ± 3.1	12.45 ± 1.6	0.10
Computer use (hours/day)	7.5 ± 4.3	6.4 ± 3.6	0.20
BMI (kg/m^2^)	25.31 ± 4.16	24.67 ± 4.56	0.51
Urea (mg/dL)	28.44 ± 5.4	30.41 ± 5.12	0.09
Creatinine (mg/dL)	0.84 ± 0.19	0.83 ± 0.20	0.82
Triglycerides (mg/dL)	208.4 ± 103.1	210.70 ± 106.4	0.92
Total cholesterol (mg/dL)	155.1 ± 39.4	152.7 ± 42.9	0.79
HDL cholesterol (mg/dL)	36.1 ± 10.1	38.5 ± 9.6	0.27
LDL cholesterol (mg/dL)	79.9 ± 28.5	75.8 ± 30.2	0.53

**Table 5:** Genotype and allele distributions of rs3808350 in ionizing radiation-exposed healthcare workers and controls.

Model/Genotype	Case *n* (%)	Control *n* (%)	χ^2^ *p* Value	OR (95% CI)	*p* Value
AA	19 (38)	8 (22.22)	0.3068	–	–
AG	26 (52)	24 (66.66)			
GG	5 (10)	4 (11.11)			
Dominant (AG + GG vs. AA)	31 (62) vs. 19 (38)	28 (77.78) vs. 8 (22.22)	–	ref. 0.4662 (0.1736–1.167)	0.1357
Allelic (G vs. A)	36 (36) vs. 64 (64)	32 (44.44) vs. 40 (55.56)	–	ref. 0.7031 (0.3852–1.280)	0.3330

Genotype and allele frequencies are presented as number (percentage). Genotype distributions (3 × 2) were compared using the chi-square (χ^2^) test or Fisher’s exact test, as appropriate. For the 2 × 2 dominant and allelic models, odds ratios (ORs) with 95% CIs are reported, and statistical significance was assessed using Barnard’s unconditional exact test (two-sided). An OR > 1 denotes higher odds of the variant carrier state (dominant model) or minor allele (allelic model) in the exposed group; an OR < 1 denotes the opposite. A *p* value < 0.05 was considered statistically significant.

**Table 6:** Genotype and allele distributions of rs3808351 in ionizing radiation-exposed healthcare workers and controls.

Model/Genotype	Case *n* (%)	Control *n* (%)	χ^2^ *p* Value	OR (95% CI)	*p* Value
GG	25 (50)	16 (44.44)	0.8531	–	–
GA	21 (42)	16 (44.44)			
AA	4 (8)	4 (11.11)			
Dominant (GA + AA vs. GG)	25 (50) vs. 25 (50)	20 (55.56) vs. 16 (44.44)	–	ref. 0.8000 (0.3285–1.893)	0.6319
Allelic (A vs. G)	29 (29) vs. 71 (71)	24 (33.33) vs. 48 (66.67)	–	ref. 0.8169 (0.4196–1.550)	0.6026

Genotype and allele frequencies are presented as number (percentage). Genotype distributions (3 × 2) were compared using the chi-square (χ^2^) test or Fisher’s exact test, as appropriate. For the 2 × 2 dominant and allelic models, odds ratios (ORs) with 95% CIs are reported, and statistical significance was assessed using Barnard’s unconditional exact test (two-sided). An OR > 1 denotes higher odds of the variant carrier state (dominant model) or minor allele (allelic model) in the exposed group; an OR < 1 denotes the opposite. A *p* value < 0.05 was considered statistically significant.

**Table 7:** Genotype and allele distributions of rs11544331 in ionizing radiation-exposed healthcare workers and controls.

Model/Genotype	Case *n* (%)	Control *n* (%)	χ^2^ *p* Value	OR (95% CI)	*p* Value
CC	27 (54)	28 (77.78)	0.0367	–	–
CT	19 (38)	8 (22.22)			
TT	4 (8)	0 (0)			
Dominant (CT + TT vs. CC)	23 (46) vs. 27 (54)	8 (22.22) vs. 28 (77.78)	–	ref. 2.981 (1.106–7.904)	0.0241
Allelic (T vs. C)	27 (27) vs. 73 (73)	8 (11.11) vs. 64 (88.89)	–	ref. 2.959 (1.282–6.606)	0.0110

Genotype and allele frequencies are presented as number (percentage). Genotype distributions (3 × 2) were compared using the chi-square (χ^2^) test or Fisher’s exact test, as appropriate. For the 2 × 2 dominant and allelic models, odds ratios (ORs) with 95% CIs are reported, and statistical significance was assessed using Barnard’s unconditional exact test (two-sided). An OR > 1 denotes higher odds of the variant carrier state (dominant model) or minor allele (allelic model) in the exposed group; an OR < 1 denotes the opposite. A *p* value < 0.05 was considered statistically significant.

**Table 8:** Hardy–Weinberg equilibrium analysis in the control group.

SNP	Observed Genotypes	Expected Genotypes	*p* (χ^2^)	*p* (Exact)
rs3808350	AA = 8, AG = 24, GG = 4	AA = 13.44, AG = 17.11, GG = 5.44	0.0685	0.0942
rs3808351	GG = 16, GA = 16, AA = 4	GG = 16, GA = 16, AA = 4	>0.9999	1.0000
rs11544331	CC = 28, CT = 8, TT = 0	CC = 28.44, CT = 7.11, TT = 0.44	0.7549	0.7526

**Table 9:** In silico functional and clinical annotation of the GPER1 rs11544331/p.Pro16Leu variant.

Parameter	Result
SNP ID	rs11544331
Gene	GPER1
Gene ID	ENSG00000164850
Protein	G protein-coupled estrogen receptor 1
UniProt entry	GPER1_HUMAN
UniProt accession	Q99527
Transcript	ENST00000397088/NM_001098201.3
ClinVar variant name	NM_001098201.3(GPER1):c.47C>T (p.Pro16Leu)
ClinVar Variation ID	1248609
ClinVar accession	VCV001248609.2
Cytogenetic band	7p22.3
Genomic location	NC_000007.14:g.1091775C>T; GRCh38: 7:1091775
Variant type	Single-nucleotide variant
Molecular consequence	Missense/non-synonymous variant
Codon change	CCA>CTA
Protein position	16
Amino acid substitution	p.Pro16Leu/P16L
VEP impact	Moderate
SIFT	0.19; Tolerated
PolyPhen-2	0.000; Benign
AlphaMissense	0.0743; Likely benign
CADD raw score	0.113797
CADD PHRED score	1.654
Grantham score	98
SpliceAI scores	0.000 for acceptor gain, acceptor loss, donor gain, and donor loss
ClinVar germline classification	Benign
ClinVar review status	Single submitter, criteria provided
ClinVar condition	Not provided
Global MAF	0.1389, T allele
gnomAD exomes AF	Approximately 0.207–0.228
Somatic clinical impact	No data submitted
Oncogenicity	No data submitted

SIFT: score ≥ 0.05 = tolerated; <0.05 = deleterious. PolyPhen-2: score < 0.446 = benign. CADD PHRED: score ≥ 20 = top 1% most deleterious variants. AlphaMissense: score < 0.340 = likely benign. VEP “moderate” impact indicates the molecular consequence of a missense variant and does not imply clinical pathogenicity. ClinVar classification was based on a single submitter with criteria provided. AF, allele frequency; MAF, minor allele frequency; VEP, Variant Effect Predictor; CADD, Combined Annotation-Dependent Depletion.

## Data Availability

The datasets used and/or analyzed during the current study are available from the corresponding author upon reasonable request.
